# 

**Published:** 1916-01

**Authors:** 


Contents, Page 5. Index to Advertisements, Page 6. Publicity Dept., Page 20.

Yearly Vol. 71 January 1916 Number 6

### Buffalo Medical Journal

Established 1845 by AUSTIN FLINT, M. D.

JAN. 10 1916

EDITOR AND PUBLISHER:

A. L. BENEDICT, A.M., M.D. 228 Summer Street, Buffalo, N. Y.

ASSOCIATE EDITORS:

New York State:

Grover W. Wende, M. D., Buffalo.

C. W. Hennington, B.S., M.D., Rochester

Charles Haase, M. D., Elmira.

Willis E. Ford, M. D., Utica.

Martin B. Tinker, B. S., M. D., Ithaca.

H. I. Davenport, A. M., M. D., Auburn.

Foreign

Sir William Osler, M. D., LL. D., Oxford, Eng.

Prof. P. K. Pel, M. D., Amsterdam, Holland.

Prof. C. A. Ewald, M. D., Berlin, Germany.

Rene Gaultier, M. D., Paris, France.

J. George Adami, M. D., LL.D., F. R. S. Montreal, Can.

Henry W. Hill, LL. D., Buffalo, Associate Editor in Medical Jurisprudence.

